# Non-canonical Notch Signaling Regulates Actin Remodeling in Cell Migration by Activating PI3K/AKT/Cdc42 Pathway

**DOI:** 10.3389/fphar.2019.00370

**Published:** 2019-04-16

**Authors:** Lei Liu, Lin Zhang, Shuo Zhao, Xu-Yang Zhao, Peng-Xiang Min, Ya-Dong Ma, Yue-Yuan Wang, Yan Chen, Si-Jie Tang, Yu-Jie Zhang, Jun Du, Luo Gu

**Affiliations:** ^1^Department of Biochemistry and Molecular Biology, Nanjing Medical University, Nanjing, China; ^2^Jiangsu Key Lab of Cancer Biomarkers, Prevention and Treatment, Collaborative Innovation Center for Cancer Personalized Medicine, Nanjing Medical University, Nanjing, China; ^3^Department of Physiology, Xuzhou Medical University, Xuzhou, China; ^4^Department of Physiology, Nanjing Medical University, Nanjing, China

**Keywords:** non-canonical Notch, PI3K/AKT, Cdc42, actin cytoskeleton, membrane protrusions

## Abstract

Tumor cell migration is a critical step in cancer metastasis. Over-activated Notch pathway can promote the migration of cancer cells, especially in the breast cancer. However, the underlying mechanism of non-canonical Notch signaling in modulating the migration has not yet been clearly characterized. Here we demonstrated that DAPT, a gamma secretase inhibitor, inhibited protrusion formation and cell motility, and then reduced the migration of triple-negative breast cancer cells, through increasing the activity of Cdc42 by non-canonical Notch pathway. Phosphorylation of AKT on S473 was surprisingly increased when Notch signaling was inhibited by DAPT. Inhibition of PI3K and AKT by LY294002 and MK2206, respectively, or knockdown of AKT expression by siRNA blocked DAPT-induced activation of Cdc42. Moreover, immunofluorescence staining further showed that DAPT treatment reduced the formation of lamellipodia and induced actin cytoskeleton remodeling. Taken together, these results indicated that DAPT inhibited Notch signaling and consequently activated PI3K/AKT/Cdc42 signaling by non-canonical pathway, facilitated the formation of filopodia and inhibited the assembly of lamellipodia, and finally resulted in the decrease of migration activity of breast cancer cells.

## Introduction

Tumor cell migration (or motility) is a critical step in cancer metastasis (Paul et al., 2017). We all know that actin cytoskeleton plays a pivot role in cell migration, which needed the coordinated turnover and remodeling of actin filaments (Shekhar et al., 2016). Cell migration can be divided into four steps: protrusion, adhesion, contraction and retraction, the forming of migration is often studied in a two-dimensional pattern, and membrane protrusions are generated at the leading edge of the cells where actin cytoskeleton remodeling provides the motile force (Fife et al., 2014). Actin-based filopodial and lamellipodial structures of cytoskeleton are important structures of cell protrusion when cells respond to extracellular signals, such as chemoattractant, and move toward on the extracellular matrix (Rauhala et al., 2013).

In cells, actin exists in monomeric (G-actin) and polymeric (F-actin) states (Fife et al., 2014). Lamellipodial and filopodial structures are the F-actin in different forms, which are the important phenotype of cell membrane protrusions, and affect cellular migration efficiency (Stricker et al., 2010). The assembly and organization of the lamellipodia and filopodia were controlled by Rho GTPases, Rac1 and Cdc42, respectively (Algayadh et al., 2016). Rac1 reorganizes the actin cytoskeleton and forms large, plate-like protrusions called lamellipodia, providing cell motility in many cell types; and Cdc42 initiates the formation of spikes-like filopodia to sense extracellular environment and guides the direction of cell migration (Fife et al., 2014). The existence of competition between different actin assembly factors for G-actin has been established recently. Actin regulators compete each other for a finite G-actin pool and therefore determine what kinds of actin structures are generated (Burke et al., 2014; Rotty and Bear, 2014). We speculate that the competition of Rac1 and Cdc42 may also control the formation of lamellipodial and filopodial structures and regulate the migration of cells.

Notch is a determinant of cell fate in mammals, which plays an important role in cell development, proliferation, differentiation, and apoptosis (Brzozowa-Zasada et al., 2017). The overexpression of Notch has been considered as cancerogenic in many human malignancies (Brzozowa-Zasada et al., 2016, 2017; Li et al., 2017). Four Notch receptor isoforms (Notch1, Notch2, Notch3, and Notch4) and five ligands (namely DLL1, DLL3, DLL4, Jag1, and Jag2) have been identified in mammals (Lamy et al., 2017; Li et al., 2017; Nowell and Radtke, 2017). It was reported that high levels of Notch1 and Jag1 are found in breast cancer patients, and it is associated with poorer overall survival (Reedijk et al., 2005; Bolós et al., 2013; Brzozowa-Zasada et al., 2016). Notch receptor-ligand binding will bring about receptor proteolysis at two distinct sites, resulting in the release of an active Notch fragment, Notch intra-cellular domain (NICD), from the plasma membrane. Importantly, the second proteolytic site of Notch is cut by the gamma secretase, and the proteolytic activity of which can be inhibited by pharmacological inhibitor, reducing the activation of Notch effectively (Brzozowa-Zasada et al., 2016; Li et al., 2017). Recombination signal binding protein for immunoglobulin kappa J region (RBPJκ) is a DNA-binding protein and constitutes a transcriptional repressor complex in the absence of NICD (Li et al., 2012). Released NICD binds to RBPJκ/CSL (CBF-1/RBPJκ, Su (H), Lag-1) in the nuclei and then activates the downstream target genes, and this process is known as canonical Notch pathway (Lamy et al., 2017). Activation of canonical Notch signaling can regulate the tumor metastasis (Brzozowa-Zasada et al., 2016). However, the canonical process that NICD induced transcription of downstream target genes and the following changes of related protein expression to realize its biological function needs a long time. Miraculously, there is also a non-canonical Notch pathway that RBPJκ/CSL are not required in the activation of Notch signaling (Ayaz and Osborne, 2014; Li et al., 2017). Reportedly, non-canonical Notch signaling interacts with PI3K/AKT, Wnt, HIF-1a or mTORC2 to regulate biological processes such as cell survival, metabolism, and differentiation (Song and Giniger, 2011; Andersen et al., 2012; Ayaz and Osborne, 2014; Polacheck et al., 2017). But there are almost no reports about the regulation of non-canonical Notch signaling on migration. To address how Notch signaling modulates the migration, we explore the effect of Notch inhibition on the small G protein dependent actin remodeling in breast cancer migration, especially in relation to the formation of filopodia and/or lamellipodia.

PI3K/AKT signaling is one of the signal pathways that can cross-talk with Notch signal pathways to precisely regulate cell behavior (Li et al., 2017). In glioma cells, Notch1 activation can induce AKT phosphorylation, facilitating the migration, and invasion (Zhang et al., 2012). Suppression of Notch1 signaling can decrease cell proliferation, migration and invasion by reducing AKT activity in breast cancer cells (Li et al., 2016). PI3K/AKT signaling is modulated by canonical and non-canonical Notch signal pathways. Here, we show that Notch inhibitor, DAPT, can depress the migration of triple-negative breast cancer cells through non-canonical pathway. However, it is surprising that phosphorylation of AKT on S473 was increased in breast cancer cells treated with DAPT. AKT phosphorylation-induced Cdc42 activation stimulates the formation of filopodial and inhibits the formation of lamellipodia, resulting in the reduction of cell migration.

## Materials and Methods

### Reagents and Antibodies

DAPT and ML141 (Cdc42 inhibitor) were purchased from Selleck Chemicals (Houston, TX, USA), MK2206 (AKT inhibitor) and LY294002 (PI3K inhibitor) were purchased from ApexBio (Houston, TX, USA) and Sigma-Aldrich (St. Louis, MO, USA), respectively. Rabbit monoclonal antibodies against phospho-AKT (308), phospho-AKT (473), AKT, Cdc42, cleaved Notch1 and WAVE2 were purchased from Cell Signaling Technology (Danvers, MA, USA). Mouse monoclonal antibody against Rac1 was purchased from BD Biosciences (San Jose, CA, USA). Rabbit monoclonal antibody against GAPDH was purchased from Bioworld Biotech (Nanjing, China) and HRP-conjugated secondary antibodies were purchased from Jeckson Immunoresearch Laboratories (West Grove, PA, USA).

### Cell Lines and Cell Culture

Human breast cancer cell lines (MDA-MB-468 and MDA-MB-231) were obtained from the Cell Biology Institute of Chinese Academy of Sciences (Shanghai, China). MDA-MB-468 and MDA-MB-231 cells were cultured in Dulbecco's modified Eagle's medium (DMEM, high glucose) (Gibco, Thermo Scientific, Grand Island, NY, USA) supplemented with 10% fetal bovine serum (FBS, v/v) (Gibco) in a humidified incubator containing 5% CO_2_ at 37°C. Cells were starved in serum-free medium for 12 h, and then treated with DAPT for various time periods as indicated before harvest.

### Plasmids and siRNAs

Full-length Cdc42 cDNA was amplified from the cDNA of MDA-MB-468 cells using the following primer set, sense: 5′-GAAGATCTATGCAGACAATTAAGTGTGTTGTTG-3′ and antisense: 5′-CGGAATTCGTAGCAGCACACACCTGCGG-3′. In these primers, BglII and EcoRI restriction site sequences have been underlined. The PCR products were cloned into the pEGFP-N1 Vector (Beyotime, Nantong, China). Cdc42-T17N and Cdc42-Q61L plasmids were mutated from Cdc42-EGFP using the following primer set, sense: 5′-TGTTGGTAAAAACTGTCTCCTGATATCCTACAC-3′ and antisense: 5′-TCAGGAGACAGTTTTTACCAACAGCACCATC-3′ and sense: 5′-ACTGCAGGGCTAGAGGATTATGACAGATTACGAC-3′ and antisense: 5′-ATAATCCTCTAGCCCTGCAGTATCAAAAAGTCC-3′, respectively. Cdc42-T17N and Cdc42-Q61L plasmids were all verified by sequencing (Genscript, Nanjing, China). The cells were planted in 6-well plates and then transiently transfected with these plasmids by using FuGENE HD Transfection Reagent (Promega Corporation, Madison, WI, USA) according to the reverse transfection method provided by the manufacturer.

Duplex oligonucleotides were synthesized and purified by GenePharma Co., Ltd (Shanghai, China). The sequences of small interfering RNA (siRNA) for AKT and RBPJκ were listed in Table 1. Cells were transfected with siRNA duplexes using Lipofectamine 2000 (Invitrogen, Carlsbad, CA, USA) according to the manufacturer's instructions. After transfected with plasmid or siRNA for 36 h, the cells were starved in FBS-free DMEM for 12 h, and then treated with DAPT for the indicated time.

**Table 1:** The sequences of siRNA used in this experiment.

**siRNA**	**Code**	**Sequence**
siAKT	1#	5′-GCACUUUCGGCAAGGUGAUdTdT-3′
	2#	5′-AGGAAGUCAUCGUGGCCAAdTdT-3′
	3#	5′-UCAUGCAGCAUCGCUUCUUdTdT-3′
siRBPJκ	1#	5′-CCUCCACCUAAACGACUUAdTdT-3′
	2#	5′-GCCCACCUCCUUGUGUAUAdTdT-3′
	3#	5′-GCAUGGCACUCCCAAGAUUdTdT-3′

### Monolayer Wound-Healing Assay

Cells were cultured in DMEM for 12 h after they reached 95–100% confluence and the scratch test was performed by scraping the cell monolayer with a 0.1–10 μl pipette tip. Medium and non-adherent cells were removed, and the cells were washed twice with PBS, then new medium with or without DAPT was added. Cell images were taken at the 0 h time point using an inverted microscope (Carl Zeiss Meditec, Jena, Germany). Cells were permitted to migrate into the clean area for 12 h in an incubator. And then, cell images were taken by using the same microscope. The migration rate were calculated by the distance between the two edges of the scratch at 0 h and 12 h, and then normoalized by corresponding control group.

### Transwell Migration Assay

Transwell migration assay was performed using a 24-well cell culture insert with 8 μm pores. Breast cancer cells were harvested, washed, and suspended in DMEM. Cells (4 × 10^4^/200 μl) were seeded on the upper chamber, and then the lower chamber was filled with 600 μl DMEM with 10% FBS. After incubation for 14 h, the cells that had migrated to the lower surface were fixed, and stained with 0.1% crystal violet for 5 min. The number of stained cells on the down surface of the membrane was counted in photos taken by using an inverted microscope (Carl Zeiss Meditec).

### Quantitative Real-Time PCR (qRT-PCR)

Total RNA was isolated from the cells and reversely transcribed as described previously (Yang et al., 2014). The cDNA was then amplified with a pair of forward and reverse primers (listed in Table 2). Briefly, PCR reactions were performed using SYBR Green PCR master mix reagents on ABI 7500 Fast Real-Time PCR System (Applied Biosystems, Foster City, CA, USA). Thermal cycling was conducted at 95°C for 5 min followed by 40 cycles of PCR using the following profile: 95°C, 10 s and 60°C, 30 s.

**Table 2:** Primer sequences used for qRT-PCR.

**Gene**	**Code**	**Sequence**
AKT	1#	F: 5′-TCCTCCTCAAGAATGATGGCA-3′
		R: 5′-GTGCGTTCGATGACAGTGGT-3′
	2#	F: 5′-GTCATCGAACGCACCTTCCAT-3′
		R: 5′-AGCTTCAGGTACTCAAACTCGT-3′
RBPJκ	1#	F: 5′-CGGCCTCCACCTAAACGAC-3′
		R: 5′-TCCATCCACTGCCCATAAGAT-3′
	2#	F: 5′-AACAAATGGAACGCGATGGTT-3′
		R: 5′-GGCTGTGCAATAGTTCTTTCCTT-3′
Hes1		F: 5′-TCAACACGACACCGGATAAAC-3′
		R: 5′-GCCGCGAGCTATCTTTCTTCA-3′

### Western Blotting

The concentration of protein extraction was measured by using BCA protein assay kit (Thermo, USA), and Western blotting was performed as previously described (Duan et al., 2016). The membrane with blots was blocked with TBST plus 5% non-fat dry milk and incubated with primary antibodies overnight at 4°C. Then the membrane was incubated with HRP-conjugated secondary antibodies for 2 h at room temperature and the protein bands on the membrane were detected with ECL reagent (Millipore, Billerica, MA, USA) by ChemiDoc XRS gel imaging system (Bio-Rad, USA). Densitometry analysis was performed using Quantity One software, and band intensities were normalized to GAPDH.

### Pulldown Assay

The GST tagged CRIB (Cdc42-Rac1-interactive binding domain, GST-CRIB, a gift from Dr. James E Casanora, University of Virginia, VA, USA) was purified from BL21 bacteria and isolated by incubation with MagneGST Glutathione Particles (Promega, Madison, WI) for 30 min at 4°C. The cells were serum-starved for 12 h and treated with DAPT for indicated time (0–12 h). Then, the cells were harvested and the proteins were extracted as described previously (Deng et al., 2016). Cdc42-GTP or Rac1-GTP bound to GST-CRIB was detected by Western blotting with anti-Cdc42 or anti-Rac1 antibody.

### Immunofluorescence Staining

Cells were plated on coverslips and then treated with DAPT for the indicated time after serum starvation overnight. Then the coverslips were treated as previously described (Duan et al., 2016). In Brief, coverslips were washed with phosphate-buffered saline (PBS), fixed with ice-cold 4% paraformaldehyde for 20 min at room temperature, and permeabilized with 0.1% Triton X-100 before blocking in 1% BSA for 1 h at room temperature. The cells were incubated with primary antibody of WAVE2 (1:100) at 4°C overnight, and then incubated with FITC-Phalloidin (1:100) and secondary antibody (1:200) for 1 h at room temperature. DAPI Fluoromount G (Southern Biotech, Birmingham, AL) were used to stain the nuclei. Images were taken by using an Olympus BX51 microscope coupled with an Olympus DP70 digital camera.

### Live Cell Imaging System

Cells were planted on 35 mm dishes using DMEM with 10% FBS and then transferred into L15 media (Wisent Bioproducts, St. Bruno, PQ, Canada) 2 h prior to imaging. Time-series images were acquired at 15 min intervals with 20 z-planes separated by 4 μm for 12 h. Imaging was performed on a heated stage and objective at 37°C by using an Andor Revolution spinning disk confocal workstation (Oxford instruments, Belfast, United Kingdom).

### Statistical Analysis

Student's *t*-test and one-way ANOVA were used to analyze differences between groups by using the SPSS statistical software program (Version 19.0; SPSS, Chicago, IL, USA). Data were presented as mean ± S.E.M. Values of *P* < 0.05 were considered statistically significant. All experiments were repeated at least three times.

## Results

### DAPT Inhibits Notch Activation and Reduces Migration in Breast Cancer Cell by Non-canonical Notch Pathway

**Figure 1:**
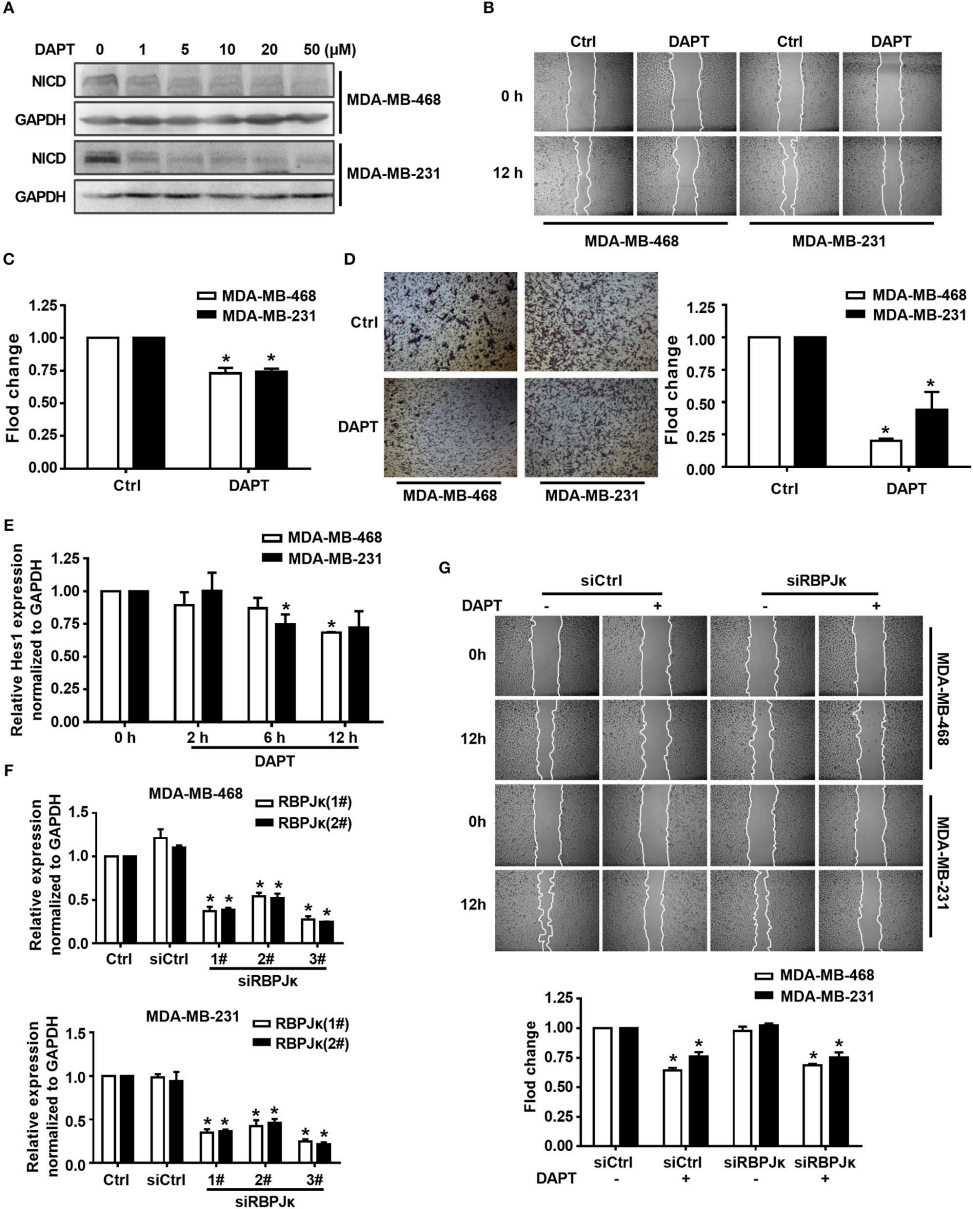
Inhibition of Notch by DAPT reduces breast cancer migration by non-canonical Notch pathway in 12 h. **(A)** The expression of NICD was detected in MDA-MB-468 and MDA-MB-231 cells when the cells were treated with 0–50 μM DAPT. **(B)** Images were taken at 0 and 12th h after wounding by an inverted microscope. **(C)** Relative migration rate was calculated and the migration of DAPT untreated cells at 12 h was set as 100%. ^*^P < 0.05 DAPT treated cells at 12 h vs. DAPT untreated cells at 12 h. **(D)** Transwell assay was performed on control vs. 20 μM DAPT treated cells to elevate the migration of cells. Migration index of Ctrl cells at 12 h was set at 100%. ^*^P < 0.05 DAPT-treated cells vs. Ctrl cells. **(E)** The effect of DAPT on the expression of Hes1 mRNA at indicated time. ^*^P < 0.05 DAPT-treated time point vs. DAPT treated 0 h. **(F)** The effect of siRBPJκ on the expression of RBPJκ mRNA. ^*^P < 0.05 cells transfected with siRBPJκ vs. cells transfected with siCtrl. **(G)** The effect of DAPT on the migration of cells transfected with siCtrl or siRBPJκ. ^*^P < 0.05 DAPT-treated cells vs. DAPT-untreated cells.

Overexpression of Notch can induce the migration of breast cancer cells while using siRNA to knock down the expression of Notch1 can reduce migration and invasion of breast cancer cells (Wang et al., 2011). However, the precise molecular mechanism of Notch in regulating cell migration remains to be elucidated. Triple-negative breast cancer is especially more aggressive in breast cancer due to their frequent recurrence and high metastatic potential (Chaudhary et al., 2014). In this study, triple-negative breast cancer cell lines MDA-MB-468 and MDA-MB-231 were used to investigate the mechanism of Notch regulation on migration of breast cancer cells. We firstly tested the effect of DAPT, a gamma secretase inhibitor, on activation of Notch and migration activity in MDA-MB-468 and MDA-MB-231 cells. The results showed that DAPT significantly inhibited the release of the NICD in MDA-MB-468 and MDA-MB-231 cells, and treatment with 20 μM DAPT for 12 h obviously reduced the migration of these cells (Figures 1A–C). The results also showed that DAPT treatment significantly reduced the motility of breast cancer cells (Figure 1D). These results were coincident with the previous studies that Notch inhibition by gamma secretase inhibitor (GSI) reduced the migration of breast cancers (Bolós et al., 2013; Peng et al., 2018).

To figure out whether DAPT inhibited migration through non-canonical Notch pathway or not, we detected the mRNA expression of Hes1, a target gene of canonical Notch signal pathway (Saha et al., 2017), by using qRT-PCR. The results showed that the mRNA expression of Hes1 did not change after DAPT treatment for 2 h, and only slight decreased after DAPT treatment for 6 h and 12 h in MDA-MB-468 and MDA-MB-231 cells (Figure 1E). The results also showed that siRNA of RBPJκ (siRBPJκ) obviously reduced the mRNA expression of RBPJκ but did not affect cell migration and did not abolish the effect of DAPT on the migration in breast cancer cells (Figures 1F,G). All these results implied that DAPT inhibited the migration of breast cancer cell through non-canonical Notch pathway.

### DAPT Inhibits the Migration of Breast Cancer Cells Through Activating Cdc42

**Figure 2:**
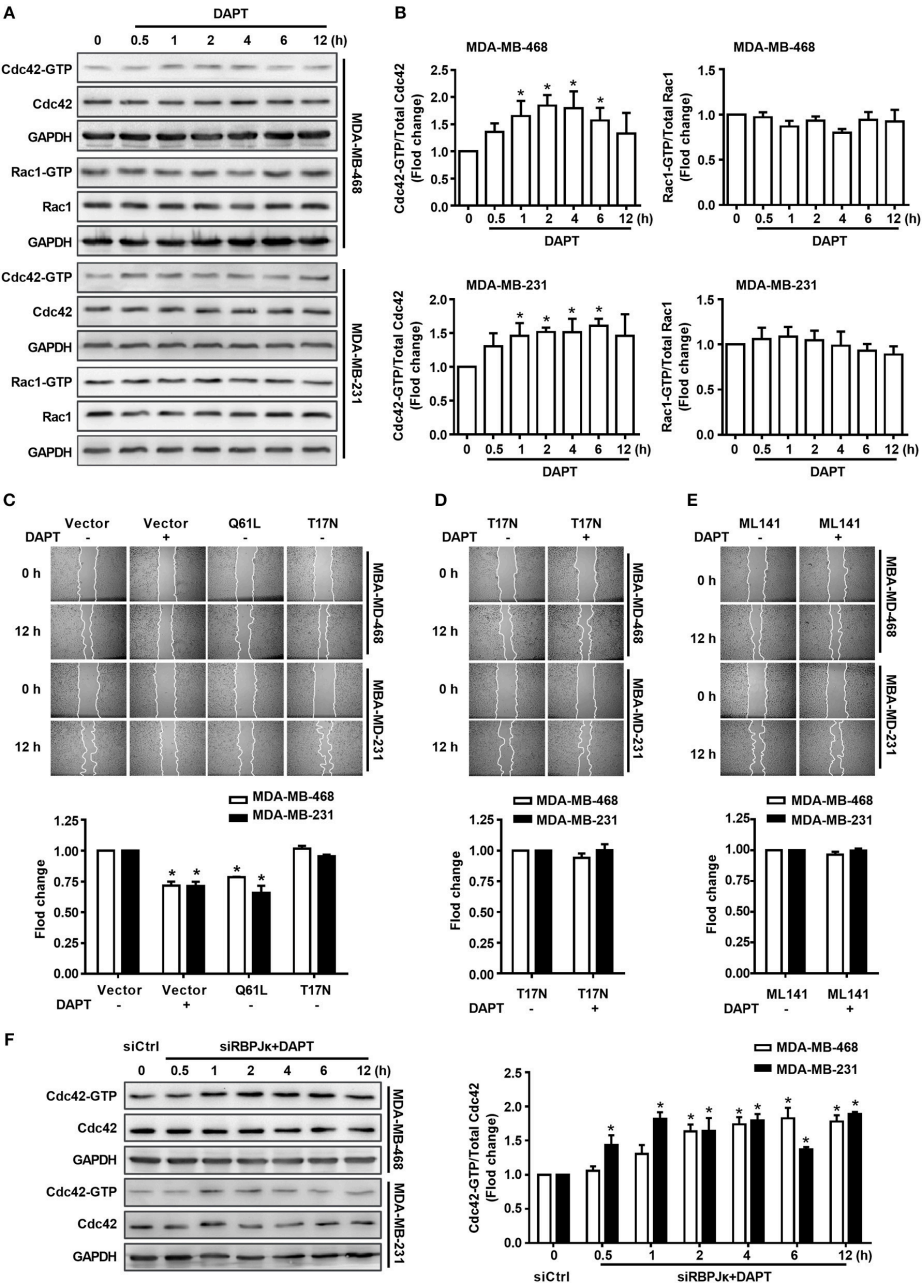
The migration of breast cancer cells was inhibited by DAPT through Cdc42 pathway. **(A,B)** The ratio of Cdc42-GTP/Cdc42 and Rac1-GTP/Rac1 were analyzed by Pulldown assay in MDA-MB-468 and MDA-MB-231 cells, which were incubated with DAPT (20 μM) for indicated time. ^*^P < 0.05 DAPT-treated time point *vs*. DAPT treated 0 h. **(C)** Effects of Cdc42-Q61L and Cdc42-T17N on the migration of cells. ^*^P < 0.05 cells transfected with plasmid *vs*. cells transfected with vector. **(D,E)** Inhibition of Cdc42-T17N or ML141 (Cdc42 inhibitor, 10 μM) on the DAPT-induced migration of MDA-MB-468 and MDA-MB-231 cells. ^*^P < 0.05 DAPT-treated cells vs. DAPT-untreated cells. **(F)** Analysis of the level of active Cdc42 in breast cancer cells transfected with siRBPJκ (3#) and then incubated with DAPT (20 μM) for indicated time. ^*^P < 0.05 cells transfected with siRBPJκ vs. cells transfected with siCtrl.

Rac1 and Cdc42 are reported to regulate the assembly and organization of the actin cytoskeleton in cell protrusions (Kato et al., 2014; Ridley, 2015). Therefore, in this study, the activity of Rac1 and Cdc42 after DAPT treatment (0–12 h) was detected in MDA-MB-468 and MDA-MB-231 cells. Panels A and B of Figure 2 showed that treatment with 20 μM DAPT increased the ratio of Cdc42-GTP/Cdc42, but the ratio of Rac1-GTP/Rac1 was not significantly changed in both MDA-MB-468 and MDA-MB-231 cells. In order to confirm whether the activation of Cdc42 participated in the inhibition of migration induced by DAPT, we constructed two GTPase defective Cdc42 mutants, Cdc42-Q61L, and Cdc42-T17N. Cdc42-Q61L was a constitutively active mutant of Cdc42 with a defect in hydrolyzing GTP. Cdc42-T17N, with a reduced affinity for nucleotides, was a dominant negative mutant of Cdc42(Ridley, 2001; Zhang et al., 2017). We found that Cdc42-Q61L inhibited the migration, but Cdc42-T17N had no effect on the migration of breast cancer cells (Figure 2C). Furthermore, the inhibition of DAPT on migration was also repressed by Cdc42-T17N or ML141 (Figures 2D,E). Moreover, Cdc42 was already activated after DAPT treatment for 2 h, but there were no obviously changes of Hes1 expression at this time (Figure 1E). Further study also showed that siRBPJκ did not inhibit the DAPT-induced activation of Cdc42 (Figure 2F). These results indicated that DAPT inhibited the migration of breast cancer cells through activating Cdc42 by non-canonical notch pathway.

### DAPT Induces S473 Phosphorylation of AKT

**Figure 3:**
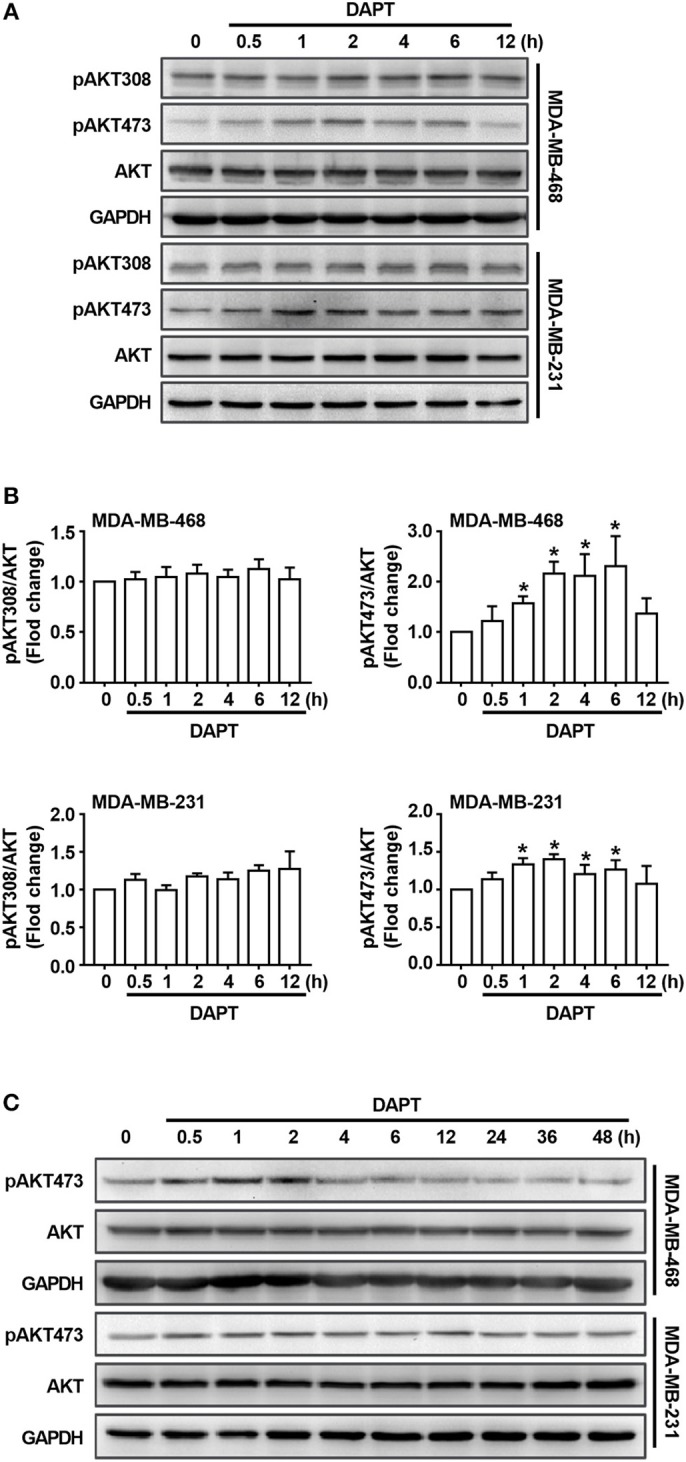
DAPT increases the level of pAKT473 in breast cancer. **(A)** The changes of the level of T308- and S473-phosphorylated AKT in cells incubated with DAPT (20 μM) for indicated time points. **(B)** Relative level of T308- and S473-phosphorylated AKT in cells incubated with DAPT for indicated time points were calculated. Phosphorylated AKT level of DAPT-untreated cells was set as 100%. ^*^P < 0.05 DAPT-treated time point vs. DAPT-treated 0 h. **(C)** The changes of the level of S473-phosphorylated AKT in MDA-MB-468 and MDA-MB-231 cells incubated with DAPT (0–48 h).

As described in the previous studies, Notch is an important regulator/modulator of PI3K/AKT signaling (Zhang et al., 2012; Hales et al., 2014; Mendes et al., 2016), while PI3K/AKT signaling plays important role in regulating activity of Cdc42(Larson et al., 2010; Tian et al., 2018). To explore the mechanism for the regulation of DAPT on Cdc42 activity, the levels of phosphorylated AKT were detected. The results showed that there was no obvious change of the level of T308-phosphorylated AKT (pAKT308) when Notch activation was inhibited by DAPT. Interestingly, we found that DAPT treatment could increase the S473 phosphorylation on AKT (pAKT473) (Figures 3A,B). However, as reported in the previous studies, S473 phosphorylation on AKT automatically increased in NICD activated cells at the 72th h after transfection (Zhang et al., 2012; Wang et al., 2014), and suppression of Notch1 downregulated AKT phosphorylation/activation in breast cancer cells at 48th h after transfection (Li et al., 2016). So, we prolonged DAPT treatment and found that the level of pAKT473 was decreased when the treatment time was longer than 24 h (Figure 3C). The results indicated that the DAPT might have different effect on AKT activation at different treatment time.

### DAPT Increases the Ratio of Cdc42-GTP/Cdc42 by Activating AKT

**Figure 4:**
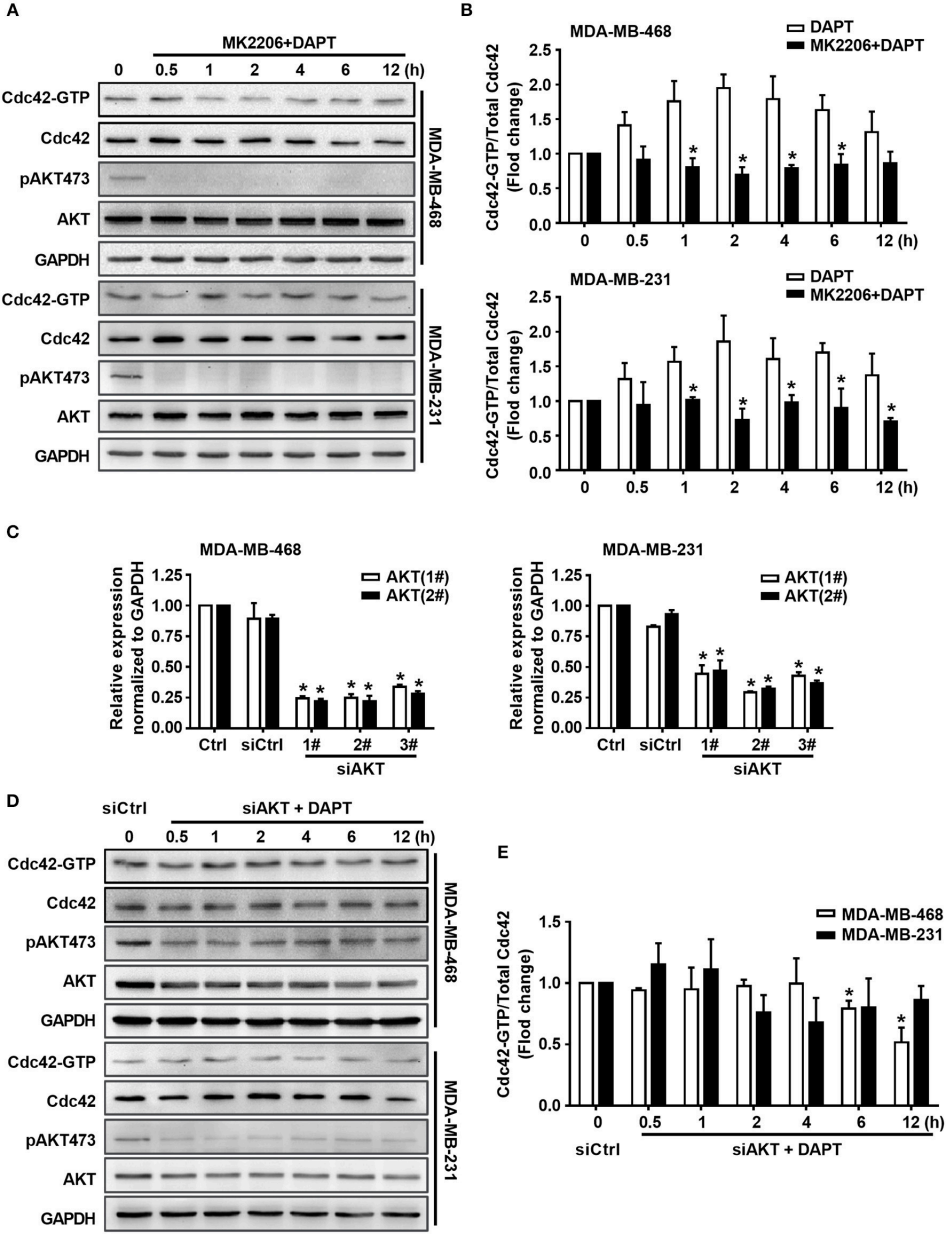
DAPT increases the ratio of Cdc42-GTP/Cdc42 by activating AKT. **(A)** The activity of Cdc42 was analyzed by Pulldown assay in MDA-MB-468 and MDA-MB-231 cells when the cells were treated with MK2206 (AKT inhibitor, 5 μM) 30 min before administration DAPT (20 μM) for indicated time points. **(B)** The quantification of the ratio of Cdc42-GTP/Cdc42 was performed when the cells were treated or untreated with MK2206 before administration DAPT for indicated time. ^*^P < 0.05 MK2206-treated cells vs. MK2206-untreated cells at corresponding time point. **(C)** The effect of siAKT on the expression of AKT. ^*^P < 0.05 cells transfected with siAKT vs. cells transfected with siCtrl. **(D,E)** The activity of Cdc42 and quantification of the ratio of Cdc42-GTP/Cdc42 in breast cancer cells transfected with siAKT (1#+2#) and then incubated with DAPT (20 μM) for indicated time points were analyzed. ^*^P < 0.05 cells transfected with siAKT (1#+2#) vs. cells transfected with siCtrl.

To elucidate whether DAPT induced activation of Cdc42 through AKT pathway or not, MK2206, an AKT inhibitor, was used to inhibit the phosphorylation of AKT. The results showed that MK2206 decreased the level of pAKT473, and inhibited DAPT induced increase in the ratio of Cdc42-GTP/Cdc42 (Figures 4A,B, Figure S1). siRNA of AKT (siAKT) was also used to decrease the expression of AKT. The results showed that the expression of AKT and pAKT473 level were significantly decreased by siAKT transfection, and the DAPT-induced increase in the Cdc42-GTP/Cdc42 ratio was reversed at the same time (Figures 4C–E). All these results supported that Notch inhibitor, DAPT, increased the activation of Cdc42 through AKT-mediated signal pathway.

### DAPT-induced AKT Activation Is Related to PI3K

**Figure 5:**
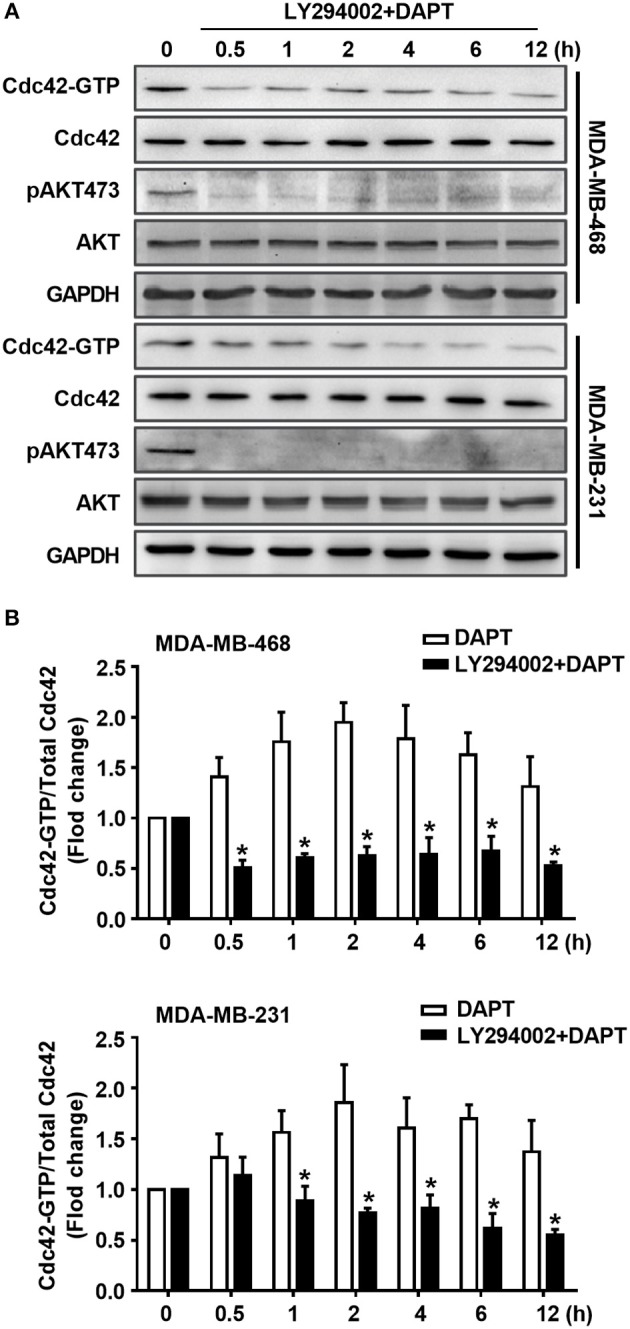
DAPT activates Cdc42 through PI3K/AKT pathway in MDA-MB-231 and MDA-MB-468 cells. **(A)** The activity of Cdc42 was analyzed by Pulldown assay in MDA-MB-468 and MDA-MB-231 cells when the cells were treated with LY294002 (PI3K inhibitor, 10 μM) for 30 min before administration DAPT (20 μM) for indicated time. **(B)** The quantification of the ratio of Cdc42-GTP/Cdc42 was performed when the cells were treated or untreated with LY294002 before administration DAPT for indicated time. ^*^P < 0.05 LY294002 treated cells vs. LY294002 untreated cells at corresponding time point.

It is well known that AKT is the downstream component of PI3K signaling. Therefore, the role of PI3K in DAPT-induced Cdc42 activation was investigated sequentially. The effect of LY294002, a PI3K inhibitor, on Cdc42 activation was tested. The results showed that LY294002 blocked the DAPT-induced phosphorylation of AKT and reduced the DAPT-increased ratio of Cdc42-GTP/Cdc42 (Figure 5, Figure S1), suggesting that DAPT activated Cdc42 through PI3K/AKT signal pathway in breast cancer cells.

### DAPT Remodels F-actin and Inhibits the Formation of Lamellipodia During Migration of Breast Cancer Cells

**Figure 6:**
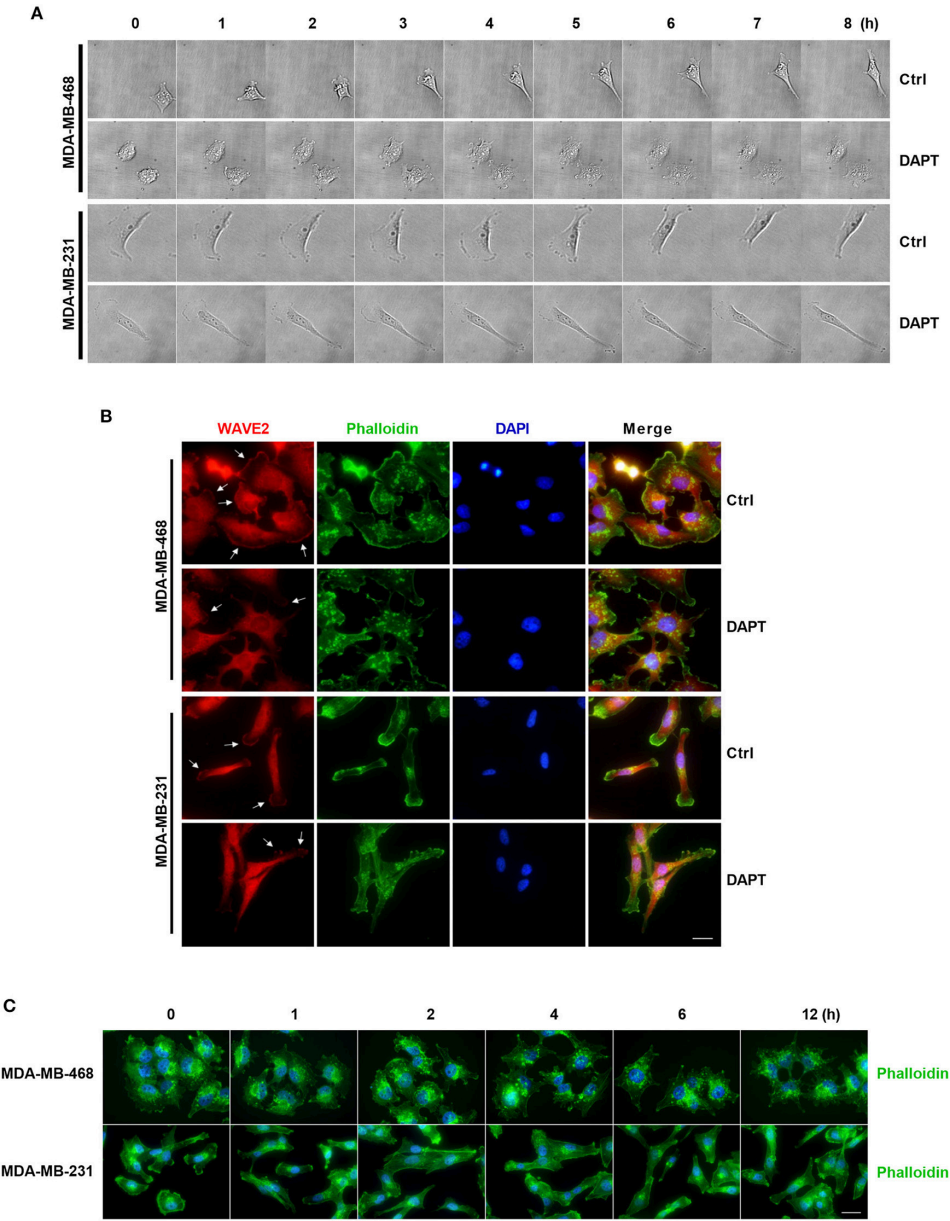
DAPT inhibits the formation of lamellipodia and induces the F-actin remodeling during migration of breast cancer cells. **(A)** The changes of protrusion formation and the cell motility of DAPT-treated or DAPT-untreated breast cancer cells were observed by live cell imaging system. **(B)** Immunofluorescence staining showing that the distribution of WAVE2 was changed by treating breast cancer cells with DAPT. White arrows represent WAVE2 aggregation under the membrane. Scale bar, 20 μM. **(C)** DAPT induced the F-actin remodeling and changed cell morphology in MDA-MB-468 and MDA-MB-231 cells. Scale bar, 20 μM.

In this experiment, the morphologic changes of the migrating cells treated or untreated with DAPT were also observed by Live cell imaging system. The results showed that in untreated cells, spike-like protrusions extended initially and then new plate-like protrusions formed on the base of spike-like protrusions. The cooperation between spike-like protrusions and plate-like protrusions at the leading edge helped to bring about the efficient migration of the cells. DAPT treatment significantly inhibited the formation of new plate-like protrusions on the base of spike-like protrusions and decreased the motility of the breast cancer cells (Figure 6A and Movies S1–S4). To confirm the F-actin structure of plate-like protrusions, immunofluorescence staining was used to detect the distribution of WAVE2, which was downstream component of Rac and crucial for the formation of lamellipodia (Takenawa and Suetsugu, 2007; Yamaguchi and Condeelis, 2007). The results showed that there were WAVE2 distribution and thickened F-actin assembling at the same membrane in DAPT-untreated cells. In DAPT-treated cells, WAVE2 distribution, and thickened F-actin assembling at membrane were decreased, indicating that the formation of lamellipodia was inhibited by DAPT treatment (Figure 6B). Immunofluorescence staining was further used to detect DAPT-induced actin cytoskeleton remodeling. The results showed that, especially in MDA-MB-468 cells, more spike-like F-actin were formed at cell edge when the cells were treated with DAPT for 4 h, resulting in morphological changes. At the same time, plate-like F-actin at cell edge were also obviously decreased in both MDA-MB-468 and MDA-MB-231 cells (Figure 6C). These results implied that DAPT remodeled the F-actin cytoskeleton and inhibited the formation of lamellipodia in cell migration, very likely, through inhibiting non-canonical Notch signaling.

## Discussion

Notch is a determinant of cell fate and its overexpression has been depicted as oncogenic in a lot of human malignancies (Li et al., 2017). Aberrant activation of Notch is associated with many tumor processes, including proliferation, malignant cell survival, angiogenesis, metastasis and epithelial-mesenchymal transition (EMT) (Li et al., 2017). In recent decades, Notch has been considered as therapeutic target of cancers, and gamma-secretase inhibitors (GSIs) are used to block the cleavage of Notch receptors at the cell membrane and inhibit the release of NICD (Brzozowa-Zasada et al., 2016). In the present study, we have shown that DAPT, a GSI, inhibits Notch signaling and then activates PI3K/AKT/Cdc42 signaling by non-canonical Notch pathway, resulting in Cdc42-mediated actin remodeling in migration of breast cancer cells.

Tumor metastasis is the major reason of poor prognosis in various types of cancers and EMT is one of the commonly accepted mechanisms of metastasis. Increasing evidence has shown that Notch is a key regulator for EMT and migration (Hassan et al., 2014; Yuan et al., 2014; Vinson et al., 2016). Through EMT, cancer cells enhance its ability of migration and invasion (Yuan et al., 2014). Activation of Notch-1 enhances cell metastasis by promoting EMT in lung cancer (Xie et al., 2012), squamous cell carcinoma (Natsuizaka et al., 2017) and breast cancer (Shao et al., 2015). Overexpression of NICD also reduces E-cadherin expression and induces EMT (Sahlgren et al., 2008). However, all the regulations of EMT are realized by canonical Notch pathway. In our experiment, the migration of breast cancer cells was effectively inhibited when the cells were treated with DAPT for 12 h. However, the EMT process and canonical Notch signaling, which process usually takes 12 h or longer, may not be suitable to explain the reduced migration at this time point.

Rho GTPases play a fundamental role in control of several biochemical processes in migration, such as cytoskeletal dynamics, integrin-mediated adhesion, and directional sensing (Fife et al., 2014; Ridley, 2015). Notch signalings were found to be closely related to Rho GTPases in many cellular biological processes. Inhibition of Rho abolishes Notch-induced phosphorylation of myosin light chain and reorganization of F-actin to adherent junctions, resulting in enhanced barrier function (Venkatesh et al., 2011). In squamous cell carcinomas, Notch1-induced RhoE activation is essential for nuclear translocation of NICD (Zhu et al., 2014). It also has been found that Notch activation induces phosphorylation of RHOGEF protein TRIO, and phosphorylated TRIO may further cause Rho activation and stimulate colorectal cancer metastasis (Sonoshita et al., 2015). These research data indicate that Notch signaling may be an important modulator of Rho GTPases. Rac1 and Cdc42 are the key members of Rho GTPases, and they can modulate the formation of membrane protrusions during tumor cell migration. Our results showed that DAPT induced the activation of Cdc42, but did not obvious change the activity of Rac1, indicating that the inhibitory effect of DAPT on migration may be associated with the activation of Cdc42 and Cdc42 related actin remodeling.

In a motile cell, polarization and extensions of actin-based protrusions occurred, forming highly motile structures called filopodia and lamellipodia, to explore the environment and migrate (Frugtniet et al., 2015). The formation of lamellipodia and filopodia are related to Rac1 and Cdc42, respectively (Frugtniet et al., 2015). Activated Rac1 activates WAVE and interacts with the Arp2/3 complex to trigger actin polymerization, forming branched actin network (Kurisu and Takenawa, 2009); while Cdc42 interacts with N-WASP and then activates Arp2/3 complex, inducing the actin polymerization at the side of existing actin filaments and the formation of spike-like projection (Nayak et al., 2013). Lamellipodial structure is thought to exert a force with variable direction in space, while lamellipodia can provide the driving force for cell migration. In contrast, filopodia can exert weaker force than lamellipodia during exploratory motion of cells (Yamaguchi and Condeelis, 2007; Yilmaz and Christofori, 2009; Stricker et al., 2010). In this paper, the results showed that DAPT activated Cdc42 but not Rac1, implying that modulation by Cdc42 on F-actin polymerization may be the reason for reduced migration in DAPT treated breast cancer cells. The results also showed that constitutively activating Cdc42-Q61L inhibited the migration, but negative mutant of Cdc42, Cdc42-T17N, had no effect on the migration of breast cancer cells. Moreover, Cdc42-T17N and Cdc42 inhibitor ML141 also blocked DAPT induce migratory inhibition. Our results also showed that Cdc42 were activated at 1h - 2h after DAPT treatment, but canonical Notch downstream signaling (indicated by expression of Hes1) did not change at this time point. These results strongly imply that DAPT-induced activation of Cdc42 may not be associated with the canonical Notch signaling. This conclusion was strengthened by experiments with the siRNA of RBPJκ. In canonical Notch signaling, when released NICD displaces the repressive cofactors and bound to RBPJκ, they would recruit a transcriptional activator complex and induce the transcription of Notch-targeted gene (Li et al., 2012). In our experiment, siRBPJκ didn't inhibit the effect of DAPT on activation of Cdc42 and migration when breast cancer cells treated with DAPT. All these results suggest that DAPT-induced Cdc42 activation is responsible for the DAPT-reduced migration by non-canonical Notch pathway.

PI3K/AKT is an important signaling pathway which regulates survival and growth in response to extracellular signals (Nitulescu et al., 2016), and phosphorylation of AKT on both S473 and T308 is required for AKT activation (Perumalsamy et al., 2009). Reportedly, Notch signaling needs to cross talk with PI3K/AKT signaling and other signaling pathways to precisely regulate cell fate (Li et al., 2017). Suppression of Notch1 activity can decrease cell proliferation, migration and invasion by attenuating PI3K/AKT/NF-κB signaling in breast cancer cells (Li et al., 2016). In glioma cells, activation of Notch1 by DLL4 stimulation or overexpression of NICD induces AKT phosphorylation, promoting the migration and invasion of the cells (Zhang et al., 2012). Notch also inhibits activation of PP2A and PTEN, induces continuous activation of PI3K/AKT, and accelerates malignant process of cancer (Hales et al., 2013; Li et al., 2016, 2017; Mendes et al., 2016). In this study, DAPT was found to activate Cdc42 through PI3K/AKT signaling pathway. When the cells were treated with DAPT, there was a transient S473 phosphorylation/activation of AKT. Interestingly, this result is opposite to the earlier reports. We further prolonged DAPT treatment time to 48 h and found that the S473 phosphorylation on AKT 24 h after DAPT treatment was decreased expectedly, possibly regulated by the canonical Notch pathway. These changed levels of S473 phosphorylation on AKT at different time points indicate that there is hysteretic regulation of Notch signals on the cell behavior. It also implies that the inhibition of DAPT on the Notch signaling and the activation of AKT may be associated with non-canonical Notch signaling. Moreover, inhibition of PI3K or AKT phosphorylation by LY294002 or MK2206, or knockdown of AKT expression by siRNA obviously inhibited the S473 phosphorylation of AKT and blocked the activation of Cdc42. All these data suggest that DAPT activates Cdc42 via PI3K/AKT signaling and then reduces the migration of breast cancer cells.

It is well known that active Cdc42 can organize actin filaments into long, parallel and tight bundles, and further cause the formation of filopodia (Svitkina et al., 2003; Stricker et al., 2010). DAPT induced activation of Cdc42 may be the reason for the remodeling of F-actin and phenotypic changes of cells. Reportedly, filopodia can reform into lamellipodia by initiating dendritic actin nucleation, and filopodia can also be formed by reorganizing a dendritic network of lamellipodia. These research data indicate that filopodia and lamellipodia are the highly interactive and inter-convertible structures (Svitkina et al., 2003; Yilmaz and Christofori, 2009). The process that actin regulates constitute specific structures of F-actin and in turn allows cells to achieve certain functions is precisely controlled in cells. Recently, it has established that the competition among different assembly factors for G-actin was existed in cells. Actin regulators compete with each other for finite G-actin and therefore determine what kinds of actin networks and structures are formed (Davidson and Wood, 2016). It is also reported that Cdc42 can bind to IRSp53, which is important molecule having a specific linker WAVE2 and Rac1 (Takenawa and Suetsugu, 2007; Kurisu and Takenawa, 2009). Bind of Cdc42 to IRSp53 decreases the affinity of IRSp53 for WAVE2 (Takenawa and Suetsugu, 2007), which will inhibit Rac1 based formation of lamellipodia. DAPT treatment significantly reduced the distribution of WAVE2 to the membrane, decreasing the formation of new plate-like protrusions and the motility of the breast cancer cells in this experiment. These data illuminate that Cdc42 activation may compete with Rac1 for finite G-actin and inhibit Rac1 based lamellipodia formation, resulting in less lamellipodial actin network assembly and migration inhibition of cells.

**Figure 7:**
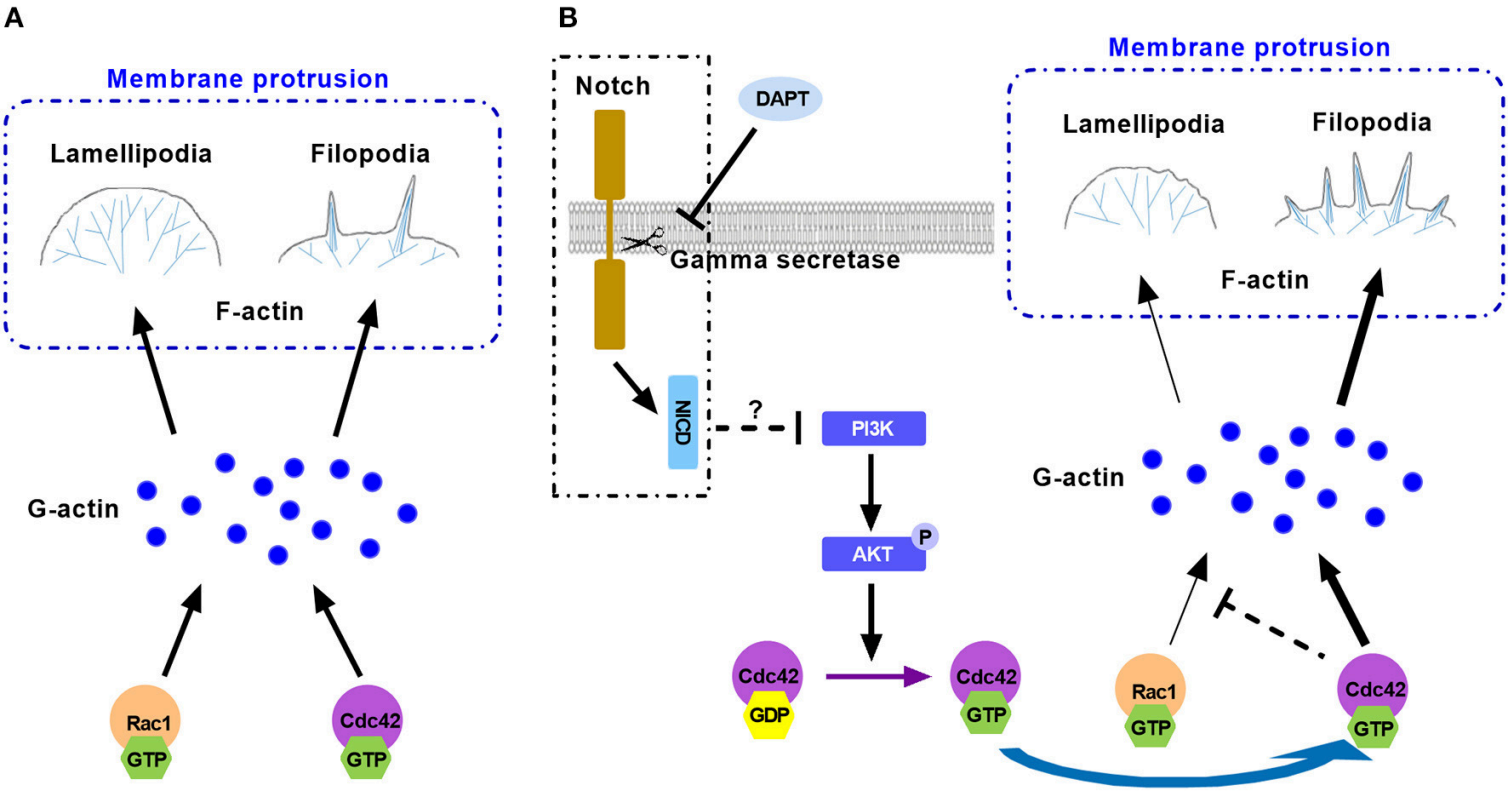
The model illustrates the mechanism for the effect of DAPT on modulating F-actin polymerization through PI3K/AKT/Cdc42 signaling by Non-canonical Notch pathway during cancer cell migration. **(A)** Rac1 and Cdc42 compete each other for G-actin to form different protrusive structures in cell migration. **(B)** DAPT activates PI3K/AKT/Cdc42 pathway through non-canonical Notch signaling. Activation of Cdc42 enhance its competition with Rac1 to bind with G-actin, which polymerizes spike-like filopodia and inhibits Rac1-based lamellipodia formation, leading to the decrease of force generation at the front of cell and the reduction of cell migration.

Taken together, Rac1 and Cdc42 are important small GTPases and they compete and cooperate with each other for finite G-actin to forming different protrusive structures in cell migration. Active Cdc42 may compete with Rac1 for finite G-actin and occupy more G-actin to form filopodia, resulting in less lamellipodial actin network assembly. Activation of Cdc42 caused more formation of filopodia and inhibited Rac1 based lamellipodia formation, providing less traction force to cell motility. This may explain the reason for the reduction of migration (Figure 7). Although we found that DAPT activated Cdc42 by PI3K/AKT signaling, the mechanism underlying the activation of PI3K/AKT signaling by non-canonical Notch is still unclear. Moreover, since many Cdc42-GEFs (guanine nucleotide exchange factors) are responsible for converting the inactive GDP-bound Cdc42 to the active GTP-bound Cdc42 (Sinha and Yang, 2008), the specific GEF which activates Cdc42 after DAPT treatment still needs to be confirmed by further experiments.

In conclusion, our research results indicate that DAPT activates PI3K/AKT/Cdc42 signaling by non-canonical Notch pathway, and the activated Cdc42 promotes the filopodia formation and inhibits lamellipodia assembly, resulting in reduced migration of breast cancer cells. The results imply that non-canonical Notch signaling may play a very important role in the rapid response of cells to the extracellular signals.

## Author Contributions

LG, JD, and LL designed the study and wrote and revised the manuscript. LL and LZ performed most of the experiments and data analysis. SZ, X-YZ, P-XM, Y-DM, Y-YW, YC, S-JT, and Y-JZ assisted in experiments and data analysis. LG supervised the experimental work. All authors read and approved the final manuscript.

### Conflict of Interest Statement

The authors declare that the research was conducted in the absence of any commercial or financial relationships that could be construed as a potential conflict of interest.

## Supplementary Material

Figure S1 The activity of Cdc42 was analyzed by Pulldown assay in MDA-MB-468 **(A)** and MDA-MB-231 **(B)** cells, which were incubated with DAPT (20 μM) for indicated time.

Movie S1 The migrating-MDA-MB-468 cell untreated with DAPT was observed at 15 min intervals for 12 h.

Movie S2 The migrating-MDA-MB-468 cell treated with DAPT was observed at 15 min intervals for 12 h.

Movie S3 The migrating-MDA-MB-231 cell untreated with DAPT was observed at 15 min intervals for 12 h.

Movie S4 The migrating-MDA-MB-231 cell treated with DAPT was observed at 15 min intervals for 12 h.
