# Supplementary figures and images for: Non-canonical Notch Signaling Regulates Actin Remodeling in Cell Migration by Activating PI3K/AKT/Cdc42 Pathway
Source: Front Pharmacol. 2019 Apr 16;10:370. doi: 10.3389/fphar.2019.00370 (PMC6477508; doi:10.3389/fphar.2019.00370)

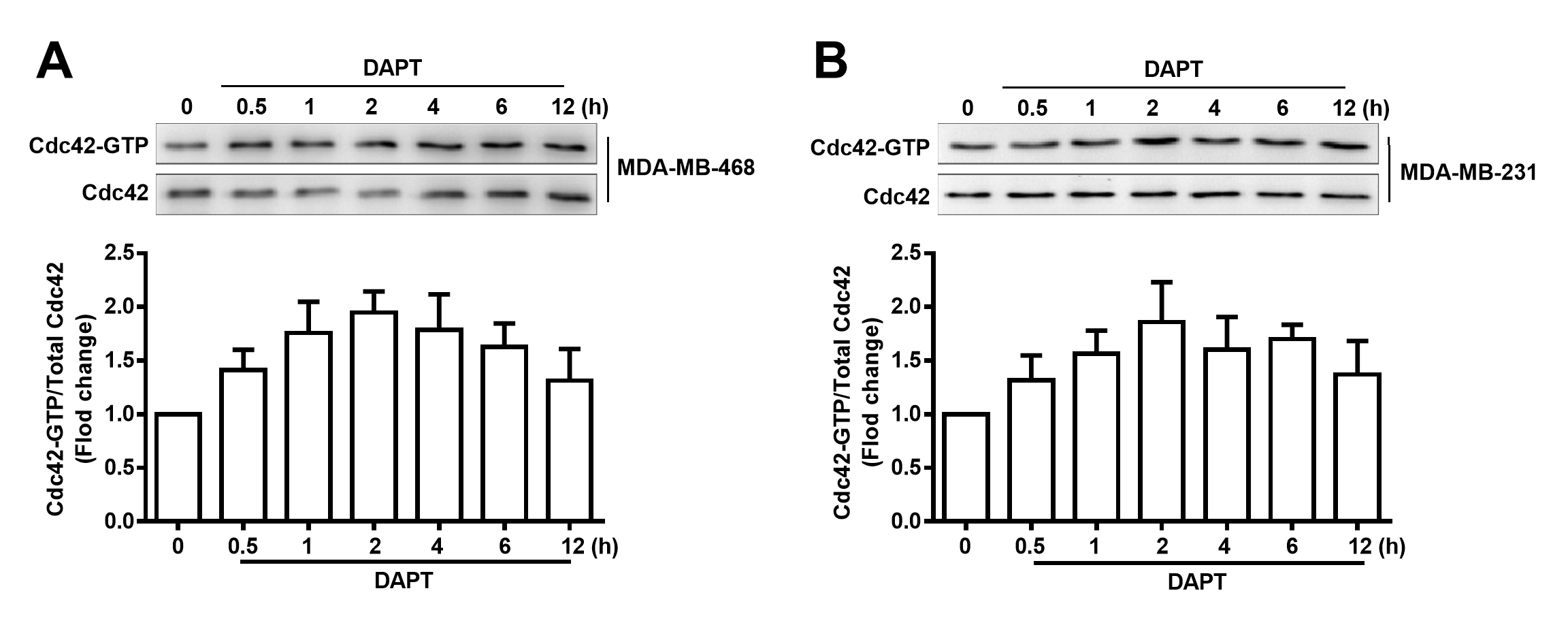

Supplement: Figure S1 — The activity of Cdc42 was analyzed by Pulldown assay in MDA-MB-468 (A) and MDA-MB-231 (B) cells, which were incubated with DAPT (20 μM) for indicated time. [file Image_1.TIF]
